# A High-Carbohydrate Diet Induces Cognitive Impairment and Promotes Amyloid Burden and Tau Phosphorylation via PI3K/Akt/GSK-3β Pathway in db/db Mice

**DOI:** 10.3390/biomedicines12081701

**Published:** 2024-07-31

**Authors:** Jialu Xu, Lei Xie, Jiaxin Yin, Xiaoli Shi, Kun Dong, Jing Tao, Weijie Xu, Delin Ma, Shujun Zhang, Juan Chen, Yan Yang

**Affiliations:** 1 Department of Endocrinology, Tongji Hospital, Tongji Medical College, Huazhong University of Science and Technology, Wuhan 430030, China; 2 Branch of National Clinical Research Center for Metabolic Diseases, Wuhan 430030, China; 3 Department of Neurosurgery, Tongji Hospital, Tongji Medical College, Huazhong University of Science and Technology, Wuhan 430030, China

**Keywords:** high-carbohydrate diet, diabetes, cognitive impairment, Alzheimer’s disease, PI3K/Akt/GSK-3β pathway

## Abstract

Background: Cognitive impairment is a prevalent complication of type 2 diabetes, influenced significantly by various dietary patterns. High-carbohydrate diets (HCDs) are commonly consumed nowadays; however, the specific impact of HCDs on cognitive function in diabetes remains unclear. Methods: The objective of this study was to investigate whether an HCD has effects on cognition in diabetes. Eight-week-old diabetic (db/db) mice and wild-type (WT) mice underwent a twelve-week dietary intervention, including a normal diet (ND), an HCD, or a high-fat diet (HFD). Following this, behavioral tests were conducted, and related hippocampal pathology was evaluated. Results: Our results demonstrated that an HCD exacerbated cognitive decline in db/db mice compared to an ND. Additionally, an HCD increased amyloid-β burden and expression of β-site APP cleaving enzyme-1. An HCD was also found to promote the phosphorylation of tau protein via the PI3K/Akt/GSK-3β pathway. Furthermore, an HCD markedly induced neuroinflammation and increased the quantity of microglia and astrocytes. However, these damages induced by an HCD were less severe than those caused by an HFD. Conclusions: Collectively, our findings indicate that a high intake of carbohydrates can have an adverse impact on cognitive function in diabetes.

## 1. Introduction

Type 2 diabetes (T2D) and cognitive impairment are prevalent chronic diseases that have a significant impact on a large proportion of the global population [1]. The global prevalence of diabetes among adults aged 20–79 years has been estimated at 10.5% [2], and individuals with diabetes are at a 1.5–2.0 times greater risk of developing cognitive decline or dementia compared to those without diabetes [3]. The incidence of dementia in patients with T2D is 83 per million person-years in people aged 60 to 64 years and more than 10 million per million person-years in people over 85 years old [4]. Diabetic cognitive dysfunction has become an increasingly common complication in individuals with diabetes.

Cognitive impairment can manifest in various degrees, ranging from mild cognitive impairment to severe forms, such as dementia [5], of which Alzheimer’s disease (AD) is the most prevalent type [6]. AD is a progressive neurodegenerative disease. Amyloid plaques, resulting from amyloid β (Aβ) accumulation and intracellular neurofibrillary tangles composed of hyperphosphorylated tau, are the hallmarks of AD pathophysiology, which also cause neuroinflammation, synaptic loss, and ultimate cognitive deficits [7,8]. These biomarkers aid in early diagnosis and monitoring of disease progression clinically. Both p-tau and t-tau levels are elevated in individuals with AD, with concentrations increasing as disease severity advances [9]. Plasma tau phosphorylated at residues threonine 181 and threonine 217 are the extensively studied blood-based AD biomarkers [10]. Studies have shown that the t-tau/Aβ42 and p-tau/Aβ42 ratios had a higher percent agreement with amyloid PET than the individual CSF markers [11]. Therapeutically, targeting amyloid and tau pathology aims to mitigate cognitive decline, though translating these discoveries into effective treatments remains challenging. Recent studies have identified neuroinflammation as the third major pathological feature of AD [12], involving astrocyte and microglia activation [13]. Neuroinflammation is now also recognized to contribute to the diffusion of tau tangles, which in turn leads to cognitive dysfunction in AD [14].

Substantial epidemiological evidence supports the close relationship between dietary patterns and diabetic cognitive impairment. Firstly, diets rich in sugar and high-glycemic-index (GI) foods can exacerbate fluctuations in blood glucose and insulin levels, worsening the occurrence of cognitive impairment [15]. Several studies have shown that hyperglycemia and elevated insulin levels, which can cause neuronal damage and death, thus affecting brain function, are important factors leading to cognitive deficits in diabetes [16,17]. Secondly, it has been found that some specific dietary patterns, such as a high-fat diet (HFD), high-cholesterol diet, and high-salt diet, can also have a detrimental effect on cognitive function [18,19,20]. These unhealthy eating habits are known to increase the risk of conditions such as high blood pressure, high cholesterol, and other diseases, further compromising brain health. Lastly, some studies have shown that certain nutrients, such as omega-3 fatty acids, vitamin B12, and β-glucan, may play a positive role in preventing or ameliorating cognitive impairment [21,22,23].

A high-carbohydrate diet (HCD) is characterized by a high intake of carbohydrates, including staple foods, pasta, desserts, and beverages. Carbohydrates play essential roles in biological systems. As energy providers, carbohydrates are broken down during digestion into glucose, which fuels cellular processes like ATP production through cellular respiration [24]. Beyond energy, carbohydrates contribute to cell structure, such as the glycan in the endoplasmic reticulum [25]. Additionally, carbohydrates serve as recognition molecules in cellular interactions, influencing processes like cell–cell communication [26]. Due to the significance of rice, pasta, and staple foods in Chinese traditional dietary culture, many people’s dietary structure is dominated by carbohydrates [27]. In addition, with the adoption of modern lifestyles, the popularity of fast food, snacks, and desserts has also led to an increased preference for an HCD [28]. Previous studies have reported the association between an HCD and obesity, nonalcoholic fatty liver disease, and other metabolic diseases [29,30], but there remains a lack of comprehensive studies exploring the association between such diets and diabetic cognitive dysfunction, thereby rendering this relationship enigmatic.

In this study, we explored the effect of an HCD on cognitive function, particularly in the context of diabetes, compared to a normal diet (ND) and an HFD, which has been observed to impair cognitive function to varying degrees. Diabetic (db/db) and wild-type (WT) control mice were subjected to an ND, HCD, or HFD, followed by cognitive behavioral tests, cognitive-related pathological analysis, and related signaling pathway exploration.

## 2. Materials and Methods

### 2.1. Animals and Experimental Design

**Figure 1:**
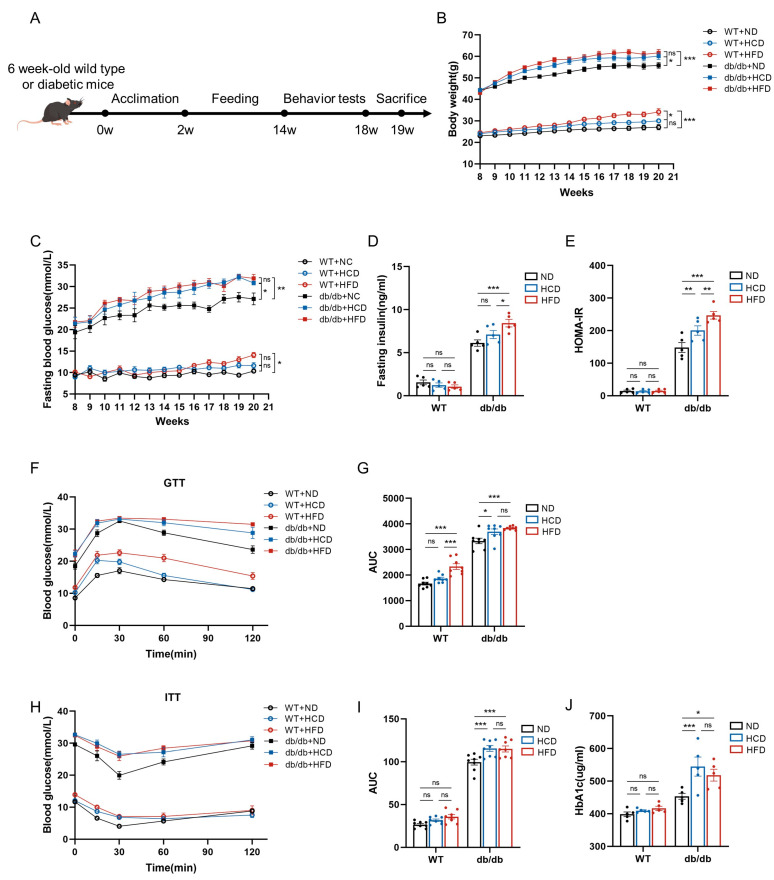
High-carbohydrate diet induces insulin resistance in db/db mice. (**A**), The experimental timeline (*n* = 10 per group); (**B**), body weight; (**C**), fasting blood glucose; (**D**), fasting insulin (*n* = 5 per group); (**E**), HOMA-IR; (**F**), glucose tolerance test (GTT) after 6 h fasting (*n* = 8 per group); (**G**), area under the curve (AUC) for the GTT after 6 h fasting; (**H**), insulin tolerance test (ITT) after 6 h fasting (*n* = 8 per group); (**I**), area under the curve (AUC) for the ITT after 6 h fasting; and (**J**), plasma level of HbA1c. Data presented as mean ± SEM. Data were analyzed with a two-way ANOVA followed by Tukey’s multiple comparisons test. * *p* < 0.05, ** *p* < 0.01, and *** *p* < 0.001; ns, not significant.

Experiments were approved by the Animal Care and Use Committee of Tongji Hospital and performed in accordance with NIH guidelines [31]. Sixty specific pathogen-free (SPF) male db/db mice (BKS-Lepr^em2Cd479^/Gpt, strain number: T002407) aged 6 weeks and sixty WT mice aged 6 weeks were obtained from GemPharmatech Co., Ltd. (Nanjing, China). Unless specifically indicated, only age-matched male mice were used in the experiments. Mice, housed four to five per cage, were allowed ad libitum access to normal food and water during the two-week acclimation period in a 12:12 light/dark cycle. Subsequently, mice were fed with either an ND (20 kcal% fat, 55 kcal% carbohydrates, and 25 kcal% protein), an HFD (60 kcal% fat, 20 kcal% carbohydrates, and 20 kcal% protein; Jiangsu Xietong Pharmaceutical Bio-engineering Co., Ltd., Nanjing, China), or an HCD (7 kcal% fat, 73 kcal% carbohydrates, and 20 kcal% protein; Jiangsu Xietong Pharmaceutical Bio-engineering Co., Ltd.) for 12 weeks (Table 1). Then, behavioral tests were carried out to evaluate cognitive ability in the eight groups before sacrifice. A visual representation of the experiment protocol is shown in Figure 1A.

### 2.2. Open Field Test

For each trial, a mouse was placed in a 45 × 45 × 45 cm^3^ square open field box and given 10 min to freely explore. The movements of the mouse were recorded using an overhead video camera. Subsequently, the data were analyzed using the VisuTrack Animal behavior analysis system (version 2.0.0, XinRuan Co., Ltd., Shanghai, China) to measure the total distance covered and velocity of each mouse. To ensure hygiene, the test area was cleaned with 75% ethanol between each trial.

### 2.3. Novel Object Recognition Test

During the initial trial, each mouse was allowed to independently examine two identical objects for 5 min within the open field area. Subsequently, they were returned to their home cages and provided with a 30 min interval. In the following trial, the mice were presented with two objects: one familiar and the other novel. They were given 5 min to explore these objects. The resulting data were expressed in terms of the frequency focused on the novel object. Frequency was calculated as [(frequency of investigating the novel object)/(total frequency of investigating both objects) × 100].

### 2.4. Barnes Maze Test

The spatial learning and memory ability was assessed using a Barnes maze, which consisted of a circular platform (100 cm in diameter) raised 90 cm above the floor. The platform was dark gray and featured 20 holes (5 cm in diameter), with 1 hole leading to an escape box. Spatial cues were placed around the platform to ensure visibility for the mice in the testing room. The simplified Barnes maze test consisted of two phases: “Spatial acquisition” and “Probe trials”. On the first day, the mice were given 5 min to familiarize themselves with the maze. Following a 30 min interval, the first acquisition session lasted for 5 min. If the mice failed to find the escape box within the allotted time, they were gently guided to it and allowed to stay there for 2 min. Over the course of the second and third days, the mice underwent two training trials (5 min per trial) with a 30 min inter-trial interval for the acquisition test. The fourth day served as a rest day for the mice. On the fifth day, a probe test was conducted, during which the escape box was removed, and the mice were given 5 min to explore the maze. The number of errors made by each mouse before locating the escape hole was recorded using a video camera and analyzed with EthoVision to evaluate spatial memory retention.

### 2.5. Glucose and Insulin Tolerance Tests

The mice were subjected to a 6 h fasting period, followed by an intraperitoneal injection of glucose (1.0 g/kg body weight, Hubei Kelun Pharmaceutical Co., Ltd., Xiantao, China) as part of the glucose tolerance test (GTT). The mice, which had undergone a 6 h fasting period, were administered an injection of human insulin (1 unit/kg Jiangsu Wanbang Biochemical Pharmaceutical Co., Ltd., Xuzhou, China) as part of the insulin tolerance test (ITT). Blood glucose concentrations were measured at 0, 15, 30, 60, and 120 min after injection using a blood glucose meter (ACCU-CHEK, Roche, Mannheim, Germany).

### 2.6. Blood and Tissue Collection

After the study, mice were euthanized over a period of 24 h. Plasma samples were collected for the measurement of glycated hemoglobin, total triglyceride (TG), and cholesterol (TC). The liver and hippocampus were harvested at the time of sacrifice, rapidly frozen in liquid nitrogen, and stored at −80 °C for further analysis.

### 2.7. Biochemical Analysis of Plasma

A glycated hemoglobin assay kit (JL20576, Shanghai Jianglai industrial Limited by Share Ltd., Shanghai, China) was used according to the manufacturer’s recommended protocols. TG and TC levels in the plasma were determined using TG assay kits and TC assay kits (A110-1-1 and A111-1-1, Nanjing Jiancheng Bioengineering Institute, Nanjing, China) according to the manufacturer’s recommended protocols. The concentrations of TG and TC were calculated using the following formula: (ODsample − ODblank)/(ODstandard − ODblank) × concentration of standards.

### 2.8. Oil Red O Staining

The liver tissues of the mice were embedded in an OTC cryostat embedding compound (Baiqiandu Technology, Wuhan, China) and subsequently frozen and sectioned (8 μm) for further analysis. Subsequently, the frozen liver sections were stained with oil red O (Baiqiandu technology, China) for 10 min, followed by washing and counterstaining with hematoxylin for 5 min. The sections were subsequently examined and evaluated under a microscope.

### 2.9. RNA Isolation and Quantitative Reverse Transcriptase-PCR (RT-qPCR)

We extracted total RNA from hippocampal samples using Trizol reagent (BS258A, Biosharp, Beijing, China), following the manufacturer’s instructions. RNA concentration and quality were assessed using a spectrophotometer (Denovix, Wilmington, DE, USA). Subsequently, we synthesized cDNA, utilizing the Hifair II Reverse Transcription System and following the manufacturer’s protocol (11141ES60, Yeasen Biotech Co. Ltd., Shanghai, China). Real-time quantitative PCR was carried out using the SYBR Green qPCR Master Mix (11202ES, Yeasen Biotech Co. Ltd., Shanghai, China) and QuantStudioTM 1 system (Thermo Fisher Scientific biosystem, Waltham, MA, USA). We normalized gene expression by glyceraldehyde 3-phosphate dehydrogenase (Gapdh), and we used the comparative CT method (2^−ΔCT^) to calculate the relative gene expression.

### 2.10. Western Blots

Frozen hippocampus samples were lysed in RIPA extract buffer (AR0102, Boster, Wuhan, China), which was chilled on ice and supplemented with protease and phosphatase inhibitors from Boster (AR1182 and AR1183, Boster, Wuhan, China). The lysates were then centrifuged at 15,000× *g* for 15 min at 4 °C to pellet cellular debris. Protein concentrations in the resulting supernatants were determined using a BCA Protein Assay Kit from Boster (AR1189, Boster, Wuhan, China). Proteins from the lysates (20 µg per sample) were resolved by 10% SDS-PAGE and electroblotted onto a PVDF membrane (IPVH00010, Millipore, Billerica, MA). The blots were then blocked with a blocking solution for 1 h at room temperature, followed by incubation with primary antibodies at 4 °C overnight. The primary antibodies used in the study were as follows: APP (A17911, ABclonal, Wuhan, China, 1:1000), BACE1 (A11533, ABclonal, Wuhan, China, 1:1000), tau5 (ab80579, Abcam, Cambridge, UK, 1:1000), phospho-tau-Ser396 (ab109390, Abcam, Cambridge, UK, 1:10,000), phospho-tau-Thr231(ab151559, Abcam, Cambridge, UK, 1:10,000), phospho-tau-Ser199 (ab81268, Abcam, Cambridge, UK, 1:10,000), PI3K p85 (4292, Cell Signaling TECHNOLOGY, Danvers, MA, USA, 1:1000), Phospho-PI3K p85-Tyr607 (AF3241, Affinity, Cincinnati, OH, USA, 1:1000), Anti-Akt1 (phospho-S473) (ab81283, Abcam, Cambridge, UK, 1:10,000), Anti-Akt1 + Akt2 + Akt3 (ab179463, Abcam, Cambridge, UK, 1:10,000), Phospho-GSK-3β(Ser9) (AP1088, ABclonal, Wuhan, China, 1:1000), GSK-3β (A11731, ABclonal, Wuhan, China, 1:1000), GFAP (ab68428, Abcam, Cambridge, UK, 1:10,000), IBA1 (ab178846, Abcam, Cambridge, UK, 1:2000), and GAPDH (Ant324, AntGene, Wuhan, China, 1:10,000). The membranes were washed in TBST buffer several times and were then incubated with secondary antibodies (SA00001-1 and SA00001-2, Proteintech, Wuhan, China) (1:5000) for 1.5 h. Finally, the membranes were visualized with an enhanced chemiluminescence detection kit (AR1171, Boster, Wuhan, China) and scanned with a GelView 6000 Pro (Antpedia, Beijing, China).

### 2.11. Enzyme-Linked Immunoassay (ELISA)

ELISAs of Aβ1-40 and Aβ1-42 were performed as per the manufacturer’s instructions (Elabscience, Wuhan, China). Briefly, frozen hippocampal tissues were homogenized in RIPA lysis buffer containing protease and phosphatase inhibitors (Boster, Wuhan, China), followed by centrifugation at 20,000× *g* for 30 min at 4 °C. The supernatants were collected as soluble fractions. The levels of Aβ1-40 and Aβ1-42 were measured using ELISA kits (E-EL-M3009 and E-EL-M3010, Elabscience, Wuhan, China) according to the manufacturer’s instructions.

### 2.12. Immunohistochemistry

Mouse brains were collected on ice immediately after sacrifice, and the right hemisphere was fixed in 4% paraformaldehyde for 48 h. The fixed brains were then embedded in paraffin and cut into 5 µm sections. Sections were deparaffinized, rehydrated, and subjected to antigen retrieval, followed by a 20 min incubation in 10% goat serum (AR1009, Boster, Wuhan, China) at room temperature. Following blocking, the sections were incubated overnight at 4 °C with a primary antibody against β-amyloid 1-42 (ab201061, Abcam, Cambridge, UK, 1:200), followed by a 45 min incubation at 37 °C with a secondary antibody (ab205718, Abcam, Cambridge, UK, 1:2000). Immunoreactivity was detected with diaminobenzidine (DAB4033, Maxim, Hong Kong, China). Finally, the sections were counterstained with hematoxylin, dehydrated, and mounted. Immunohistochemistry images were captured using a 1 microscope (Olympus CX3, Tokyo, Japan).

### 2.13. Immunofluorescence

Mouse brains were collected on ice immediately after sacrifice, and the right hemisphere was fixed in 4% paraformaldehyde for 24 h, dehydrated with 30% sucrose for 24 h, and coronally cut into 30 μm sections using a cryostat (CryoStar NX50, Thermo Fisher Scientific, Waltham, MA, USA). For immunofluorescence staining, brain sections were blocked with blocking buffer (5% BSA, 3% horse serum, and 0.3% Triton-X 100 in phosphate-buffered saline) for 1–2 h at room temperature, followed by incubation with primary antibodies overnight at 4 °C. We used the following primary antibodies: guinea pig polyclonal IBA1 (OB-PGP049-01, Biofarm, Hangzhou, China, 1:200) and rabbit monoclonal GFAP antibody (ab68428, Abcam, Cambridge, UK, 1:200). The immunofluorescent signals were labeled with fluorescent secondary antibodies as follows: donkey anti-Rabbit IgG (H + L) Highly Cross-Adsorbed Secondary Antibody, Alexa Fluor™ 594 (A21207, Thermo Fisher Scientific, Waltham, MA, USA, 1:600), and goat-anti-guinea pig IgG(H + L), 488 nm (OB-GP488-50, Biofarm, Hangzhou, China, 1:200). The images were acquired using a confocal microscope (Olympus, Tokyo, Japan).

### 2.14. Statistical Analysis

The details for each experiment are indicated in the figure legends. All data are expressed as the mean ± the standard error of the mean (SEM). Statistical analyses were performed using Microsoft Office Excel (2019) and GraphPad Prism (version 8) software. All data were tested for normal distribution using the Shapiro–Wilk test. Intergroup differences were assessed using two-way ANOVA (for more than two groups), followed by Tukey’s multiple comparisons test. Significance was set at *p* < 0.05 and expressed as * *p* < 0.05, ** *p* < 0.01, and *** *p* < 0.001.

## 3. Results

### 3.1. High-Carbohydrate Diet Induces Insulin Resistance in db/db Mice

Eight-week-old WT mice or db/db mice were subjected to a 12-week dietary intervention, including an ND, HCD, or HFD (Figure 1A). In the db/db group, mice fed an HCD exhibited similar body weights to those fed an HFD. By the end of the feeding period, both groups were heavier than the control group, and there was no significant difference in body weight between the mice fed an HCD and an HFD. In the WT group, HCD feeding did not increase body weight compared to ND feeding, whereas mice fed an HFD displayed higher body weights than the other two groups (Figure 1B).

To assess the impact of an HCD on insulin resistance, we measured fasting glucose and fasting insulin levels in both WT and db/db mice. In the db/db group, mice fed an HCD and an HFD exhibited higher fasting glucose levels than those fed an ND, with no difference between the HCD and HFD subgroups. Within the WT group, HCD consumption did not elevate fasting glucose levels compared to ND intake, while mice fed an HFD exhibited higher fasting glucose levels than the control group (Figure 1C). Analysis of fasting insulin levels revealed that, in comparison to the ND feeding, HCD feeding did not raise insulin levels in the db/db group, while the HFD-fed group exhibited elevated fasting insulin levels (Figure 1D). Consistently, the Homeostatic Model Assessment of Insulin Resistance (HOMA-IR) indicated that, in the db/db group, the mice fed an HCD showed increased insulin resistance compared to those on the ND, suggesting an exacerbation of insulin resistance by HCD consumption. Furthermore, the HFD group exhibited even more severe insulin resistance compared to the HCD group (Figure 1E).

Subsequently, we conducted a glucose tolerance test (GTT) and an insulin tolerance test (ITT). The GTT results showed that both HCD and HFD feeding impaired glucose tolerance, with no discernible difference between the HCD and HFD in the db/db group. Conversely, in the WT group, mice fed an HCD did not suffer impaired glucose tolerance (Figure 1F,G). The ITT yielded similar results. Impaired insulin tolerance was observed in mice fed on an HCD and HFD, and no difference was found between the HCD and HFD in the db/db group (Figure 1H,I). Moreover, we observed that the HCD and HFD both increased the level of plasma glycated hemoglobin A1c (HbA1c) in db/db mice, whereas they did not influence it in WT mice (Figure 1J).

### 3.2. High-Carbohydrate Diet Leads to Impaired Lipid Metabolism in db/db Mice

**Figure 2:**
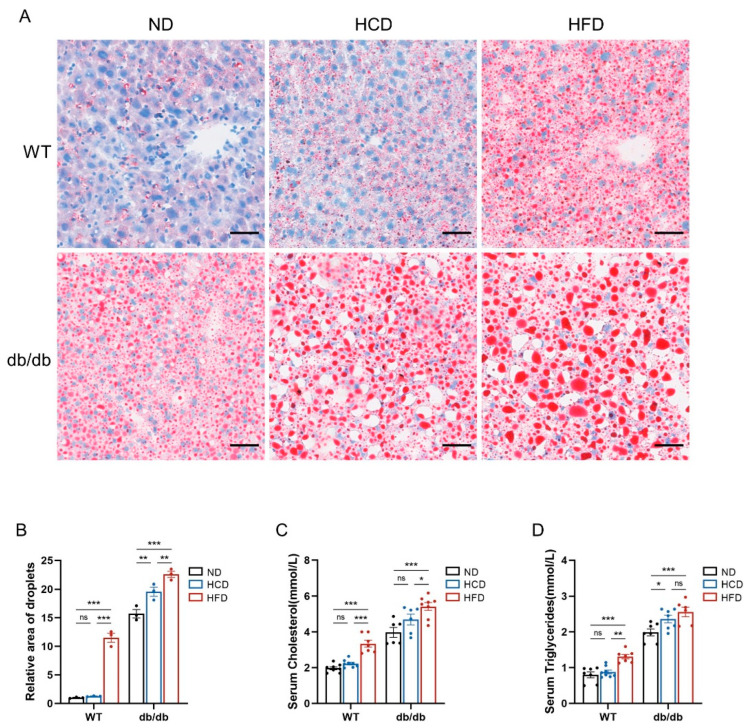
High-carbohydrate diet leads to impaired lipid metabolism in db/db mice. (**A**), Oil red O staining of mice liver in six groups (scale bar = 50 μm); (**B**), relative area of lipid droplets (*n* = 3 per group); (**C**), serum cholesterol levels (*n* = 6–8 per group); and (**D**), serum triglyceride levels (*n* = 6–8 per group). Data presented as mean ± SEM. Data were analyzed with a two-way ANOVA followed by Tukey’s multiple comparisons test. * *p* < 0.05, ** *p* < 0.01, and *** *p* < 0.001; ns, not significant.

To investigate whether an HCD influences lipid metabolism, we measured the lipid content in the liver, serum cholesterol, and serum triglycerides. Oil red O staining of liver sections showed that an HCD promoted lipid accumulation in the liver of db/db subgroups, and mice fed an HFD exhibited more severe conditions, while the lipid content in WT mice was influenced by the HFD but not the HCD (Figure 2A,B). Meanwhile, the levels of serum cholesterol and serum triglycerides were analyzed. Obviously, in the db/db group, mice fed an HFD had higher serum cholesterol levels than those on ND or HCD diets, while HCD feeding did not increase serum cholesterol compared to the ND feeding regimen. Additionally, the same effect of an HCD and HFD on serum cholesterol was observed in the WT group (Figure 2C). Conversely, the effect of the feeding regimen on serum triglycerides was slightly different. In the db/db group, the level of serum triglycerides was higher in mice fed an HCD or HFD than those fed an ND, with no significant difference between the HCD and HFD subgroups. In WT mice, HFD feeding increased serum triglycerides, but HCD feeding did not compared to ND feeding (Figure 2D).

### 3.3. High-Carbohydrate Diet Causes Cognitive Impairment in db/db Mice

**Figure 3:**
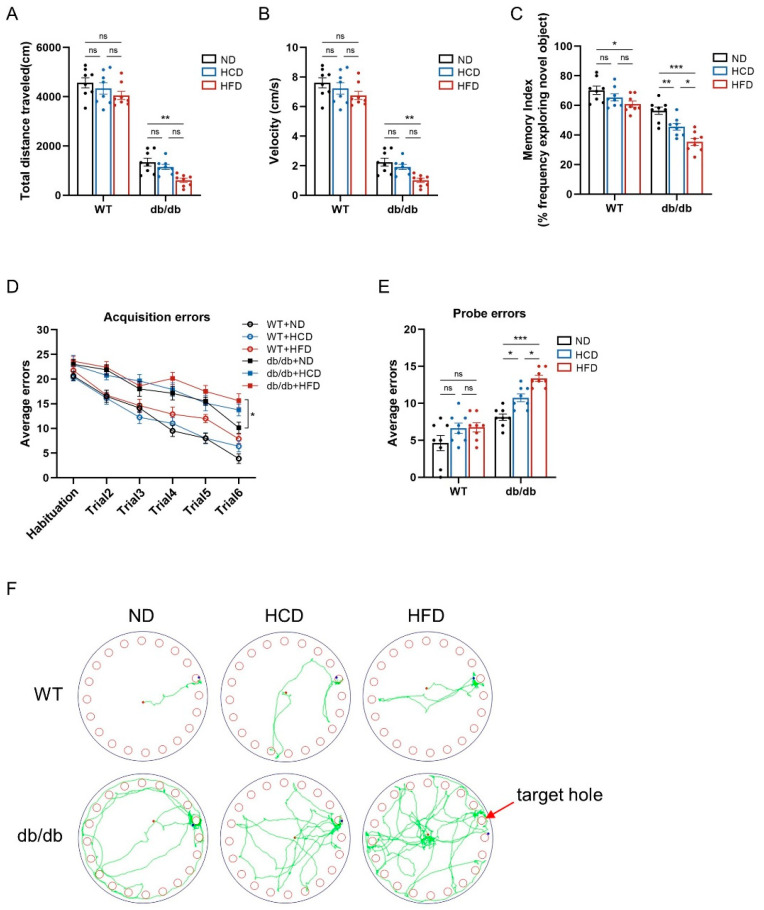
High-carbohydrate diet causes cognitive impairment in db/db mice. (**A**,**B**), The total distance traveled and average velocity in the open field test were assessed (*n* = 8 per group); (**C**), frequency of exploring the novel object in the novel object recognition test was assessed; and (**D**–**F**), acquisition errors, probe errors, and representative track images of mice in the probe trial in the Barnes maze test were assessed. Data presented as mean ± SEM. Data were analyzed with a two-way ANOVA followed by Tukey’s multiple comparisons test. * *p* < 0.05, ** *p* < 0.01, and *** *p* < 0.001; ns, not significant.

To evaluate whether long-term HCD feeding alters cognition, behavioral tests were conducted 12–14 weeks after the initiation of feeding. The open field test was utilized to examine the locomotor behaviors. The results indicated that HCD feeding did not decrease total distance and velocity in both db/db mice and WT mice, but an HFD decreased it in the db/db group (Figure 3A,B), suggesting that the HCD did not influence mice locomotivity. Meanwhile, we conducted a novel object recognition test and found that HCD feeding reduced the frequency of exploring new objects, with an HFD exacerbating this effect in the db/db group. However, in WT mice, an HCD did not influence the frequency of exploring new objects, but mice fed on an HFD had a lower frequency than the other two subgroups (Figure 3C). Subsequently, a Barnes maze test was used to detect the spatial learning and memory abilities of mice. In the Barnes maze test, mice fed on an HFD made more acquisition errors on trial 6 compared to those on an ND in the db/db group (Figure 3D). Moreover, HCD feeding increased probe errors compared to the control ND subgroup in db/db mice, with HFD feeding further exacerbating this effect. However, no significant difference was observed among the subgroups in WT mice (Figure 3E). Additionally, in db/db mice, HCD-fed mice displayed less tracking near the target hole and explored more near the error hole compared to the ND-fed mice, and the exploration track before escaping of HFD-fed mice was more complex (Figure 3F). Taken together, these findings suggested that an HCD impaired the learning and memory abilities of mice under diabetic pathological conditions.

### 3.4. High-Carbohydrate Diet Escalates Aβ Burden in Hippocampus in db/db Mice

**Figure 4:**
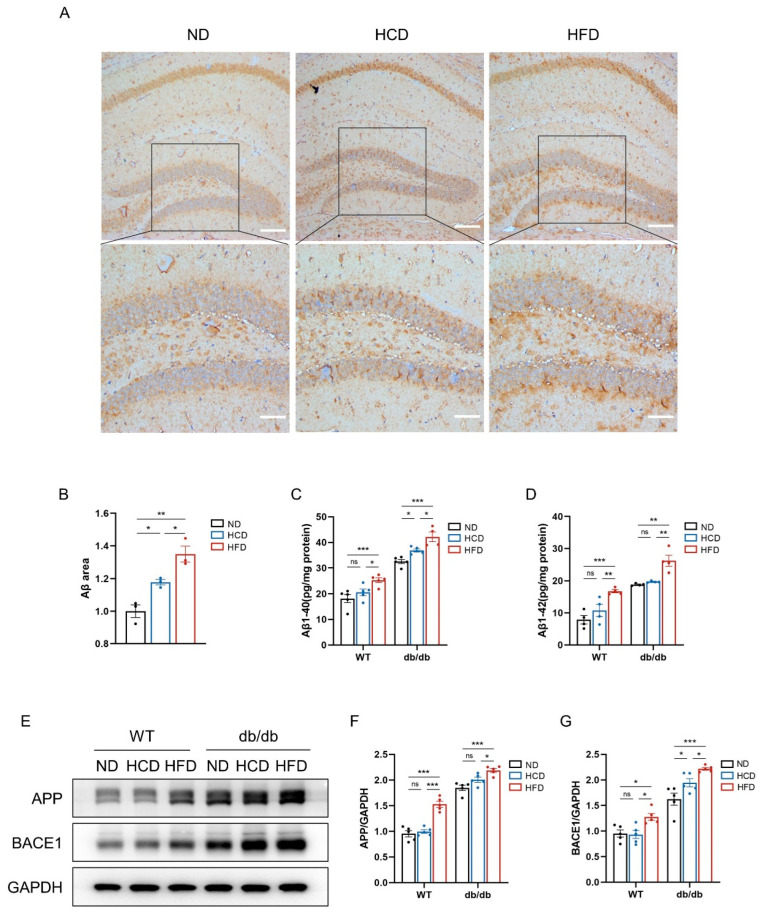
High-carbohydrate diet increases Aβ burden in hippocampus in db/db mice. (**A**), Immunostaining for Aβ plaques in the hippocampus of mice in db/db mice (scale bars = 200 mm in low-magnification and scale bars = 100 mm in high-magnification image); (**B**), relative area of Aβ plaques (*n* = 3 per group); (**C**,**D**), ELISAs of Aβ1-40 and Aβ1-42 levels in the hippocampus (*n* = 4–5 per group); (**E**), Western blots of APP and BACE1 in hippocampus of mice in six groups (*n* = 5 per group); (**F**), relative protein expression of APP; and (**G**), relative protein expression of BACE1. Data presented as mean ± SEM. Data were analyzed with a two-way ANOVA followed by Tukey’s multiple comparisons test. * *p* < 0.05, ** *p* < 0.01, and *** *p* < 0.001; ns, not significant.

To explore whether an HCD affects Aβ burden in the hippocampus of mice, immunohistochemical staining was utilized to detect Aβ plaque deposition in brain sections of both db/db mice and WT mice. The results showed that an HCD increases Aβ plaque deposition in the hippocampus of db/db mice, which was further increased by an HFD. In WT mice, an HCD did not increase Aβ deposition in the hippocampus, but an HFD did (Figure 4A,B). Subsequently, ELISA was performed to examine the levels of Aβ1-40 and Aβ1-42 fractions in the hippocampal homogenates. Consistent with our immunohistochemical staining results, it showed that an HCD elevates the level of Aβ1-40 in the hippocampus, and this effect was further enhanced by an HFD in db/db mice. But in WT mice, an HCD did not affect Aβ1-40 content in the hippocampus, while an HFD increased it (Figure 4C). Importantly, an HCD did not increase Aβ1-42 content either in the db/db or WT groups (Figure 4D). Overall, these data clearly showed that HCD feeding aggravated Aβ burden, particularly Aβ1-40 in db/db mice.

Subsequently, we performed Western blots to assess the protein level of amyloid precursor protein (APP) and β-site APP cleaving enzyme-1 (BACE1) in the hippocampus, a phenomenon associated with cognitive impairment [32,33]. In db/db mice, HCD feeding enhanced the expression of BACE1 protein compared to ND feeding, while it did not affect the expression of APP. Moreover, mice fed an HFD exhibited higher levels of APP and BACE1 than the other two subgroups. In the WT group, HCD feeding had no impact on the expression of APP or BACE1 in the hippocampus, but HFD feeding did (Figure 4E–G). These results implied that an HCD primarily influenced the BACE1 level, thereby promoting Aβ accumulation.

### 3.5. High-Carbohydrate Diet Aggravates Tau Hyperphosphorylation via PI3K/Akt/GSK-3β in Hippocampus in db/db Mice

**Figure 5:**
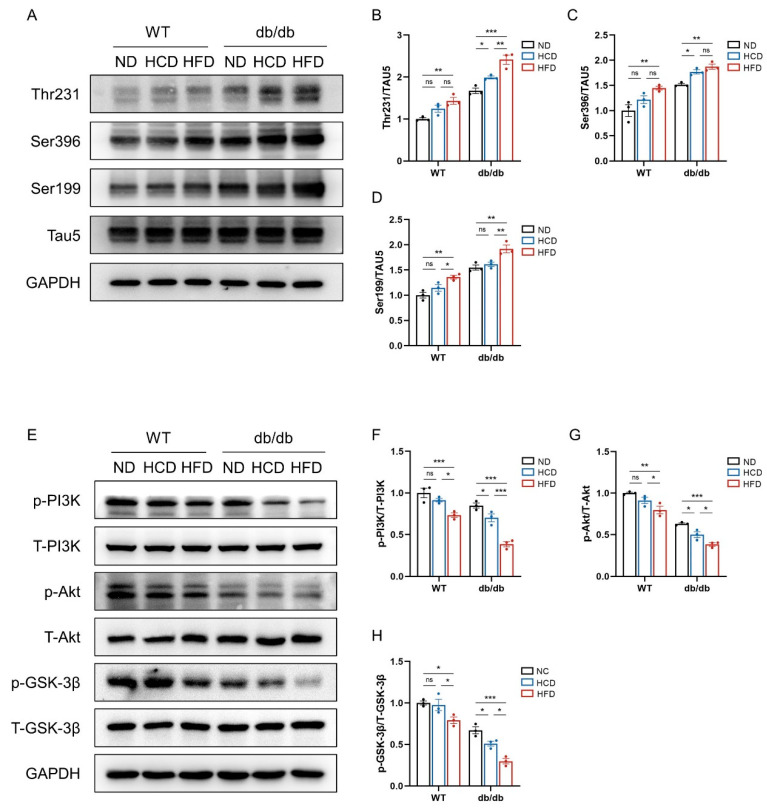
High-carbohydrate diet aggravates tau hyperphosphorylation via PI3K/Akt/GSK-3β in db/db mice. (**A**), Western blots of phosphorylation levels of tau protein (Thr231, Ser396, and Ser199 site) in the hippocampus of mice in six groups (*n* = 3 per group); (**B**), relative phosphorylation of Thr231 site of tau protein; (**C**), relative phosphorylation of Ser396 site of tau protein; (**D**), relative phosphorylation of Ser199 site of tau protein; and (**E**), Western blots of insulin signaling-related protein expression in the hippocampus of mice in the six groups (*n* = 3 per group). Phosphorylation of PI3K, AKT, and GSK3β was quantified and normalized by total PI3K, Akt, and GSK-3β, respectively. (**F**), Relative phosphorylation of PI3K; (**G**), relative phosphorylation of Akt; and (**H**), relative phosphorylation of GSK-3β. Data presented as mean ± SEM. Data were analyzed with a two-way ANOVA followed by Tukey’s multiple comparisons test. * *p* < 0.05, ** *p* < 0.01, and *** *p* < 0.001; ns, not significant.

Given that tau hyperphosphorylation is a significant pathology in AD, we investigated the influence of an HCD on the level of tau phosphorylation levels in the hippocampus. We assessed the phosphorylation levels of tau protein at the Ser396, Thr231, and Ser199 sites (Figure 5A). It was found that at the Ser396 site of tau protein, HCD-fed mice exhibited higher tau phosphorylation compared to ND-fed mice in the db/db group, and HFD-fed mice showed even greater levels of tau phosphorylation than those fed an HCD. In contrast, in the WT group, HCD feeding did not escalate tau phosphorylation in the hippocampus, but HFD feeding did (Figure 5B). Similar patterns were observed for tau phosphorylation at the Thr231 site (Figure 5C). However, the impact of an HCD on the phosphorylation of Ser199 differed. An HCD did not elevate phosphorylation levels in the hippocampus either in the WT or db/db groups compared to ND-fed mice, but an HFD increased it when compared to both ND- and HCD-fed mice (Figure 5D). These results indicated that an HCD generally increased the phosphorylation levels of tau protein in the hippocampus of db/db mice.

The increased phosphorylation of tau in the hippocampus suggested altered activity of tau kinases. Glycogen synthase kinase-3β (GSK-3β) is a major kinase responsible for tau hyperphosphorylation and impairs memory in AD. Several studies have illustrated the involvement of the activated phosphatidylinositol 3-kinase/protein kinase B (PI3K/Akt)/GSK-3β signaling pathway in AD neuropathology as a potential mechanism [34,35]. Therefore, we hypothesized that an HCD would increase the phosphorylation levels of the PI3K/Akt/GSK-3β (Ser9) signaling pathway, resulting in hyperphosphorylation of tau protein (Figure 5E). As the results showed in the db/db group, phosphorylation of PI3K and Akt in the HCD subgroup was suppressed compared to the ND subgroup, and this effect was further increased by an HFD compared to the HCD subgroup. In the WT group, an HCD did not decrease PI3K and Akt phosphorylation (Figure 5F,G). Consistently, in the hippocampus of db/db mice, the phosphorylation of GSK-3β (Ser9) in the HCD subgroup was lower than in the ND subgroup, resulting in an activation effect of GSK-3β, and an HFD further reduced it when compared to the HCD subgroup. But in the WT group, an HCD did not decrease GSK-3β (Ser9) phosphorylation (Figure 5H). An HCD suppressed the phosphorylation of Ser9 of GSK-3β, indicating the activation of GSK-3β, resulting in hyperphosphorylation of tau. Collectively, an HCD impaired the PI3K/Akt/GSK-3β signaling pathway and further promoted tau phosphorylation.

### 3.6. High-Carbohydrate Diet Increases Neuroinflammation in db/db Mice

**Figure 6:**
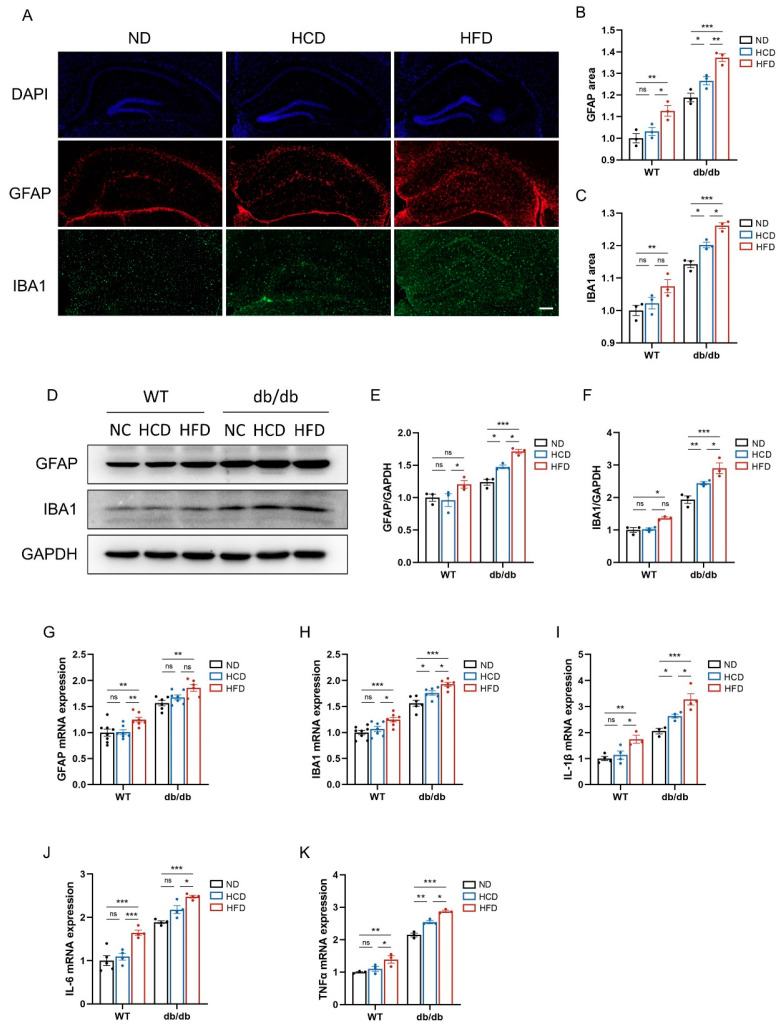
High-carbohydrate diet increases neuroinflammation in db/db mice. (**A**), Representative images of IBA1 (microglia) costained with GFAP (astrocytes) in the hippocampus of mice in the db/db group (scale bar = 200 μm); (**B**), relative area of GFAP (*n* = 3 per group); (**C**), relative area of IBA1 (*n* = 3 per group); (**D**), Western blots of GFAP and IBA1 in the hippocampus of mice in six groups (*n* = 3 per group); (**E**), relative protein expression of GFAP; and (**F**), relative protein expression of IBA1. (**G**,**H**), Hippocampal mRNA expression of GFAP and (**G**) IBA1 (**H**) normalized to GAPDH expression (*n* = 6–8 per group); (**I**–**K**), hippocampal mRNA expression of IL-1β (**I**), IL-6 (**J**), and TNFα (**K**) normalized to GAPDH expression (*n* = 4–5 per group). Data presented as mean ± SEM. Data were analyzed with a two-way ANOVA followed by Tukey’s multiple comparisons test. * *p* < 0.05, ** *p* < 0.01, and *** *p* < 0.001; ns, not significant.

Due to the chronic neuroinflammatory nature of AD and its connection to abnormal activation of glial cells, we proceeded to conduct immunofluorescence staining using antibodies targeting specific markers for microglia (ionized calcium-binding adaptor molecule 1 (IBA1) and astrocytes (glial fibrillary acidic protein (GFAP)) (Figure 6A, Figure S1). In comparison to mice fed an ND, mice fed an HCD exhibited an increased abundance of IBA1^+^ microglia and GFAP^+^ astrocytes in the hippocampus for db/db mice, with even higher levels observed in mice fed an HFD. In WT mice, an HCD did not increase the quantity of IBA1^+^ microglia and GFAP^+^ astrocytes, whereas an HFD did (Figure 6B,C). In agreement with the findings from immunofluorescence analysis, Western blots and RT-qPCR showed similar impacts of an HCD and HFD on GFAP and IBA1 (Figure 6D–H). The findings suggested that an HCD significantly elevated the expression of microglia and astrocytes in db/db mice.

In addition, we examined the gene expression of *interleukin-1β (IL-1β)*, *interleukin-6 (IL-6)*, and *tumor necrosis factor-alpha (TNF-α)* in the hippocampus. Correspondingly, an HCD upregulated the mRNA expression of *IL-1β*, *IL-6*, and *TNF-α* in db/db mice, thereby promoting hippocampal inflammation (Figure 6I–K).

## 4. Discussion

Diabetic cognitive impairment has long been a major public concern, and substantial evidence has shown a close association between diets and this condition. Given the importance of rice, noodles, and other staple foods in traditional dietary culture, HCDs are commonly practiced in China, with a substantial number of individuals adopting a carbohydrate-based dietary pattern [27]. However, limited research has investigated the impact of the HCD on the cognitive function of diabetic patients, and it remains unclear whether they improve or exacerbate cognitive impairment. In this study, we selected WT mice and db/db mice, providing them with an ND as the negative control, an HFD as the positive control, or an HCD. We examined the effects of an HCD on mouse cognition and compared the findings with those of mice fed an ND or HFD. Consistent with previous studies, our results revealed that an HFD induced cognitive impairment in both WT and db/db mice [36]. However, the impact of an HCD on cognitive function varied between the two genotypes. In db/db mice, an HCD worsened cognitive impairment compared to an ND but to a lesser extent than an HFD. In contrast, an HCD did not cause cognitive impairment in WT mice. A clinical study reported similar findings, indicating that different types of carbohydrates have diverse effects on cognitive function, showing individual variability. For individuals with normal fasting blood glucose levels, carbohydrate intake had no significant impact on memory performance [37]. Conversely, participants with impaired fasting blood glucose exhibited significantly poorer performance on a memory recall test following a carbohydrate-rich meal [37]. Another study also showed that the occurrence of cognitive impairment was 80% higher in individuals with chronically high carbohydrate intake than in controls aged 70 to 89 years [38]. It may be due to blood glucose fluctuation. Blood glucose remains stable in healthy individuals, supporting consistent energy supply and brain function [39]. Carbohydrate intake did not cause significant changes in their blood glucose levels. Conversely, individuals with impaired fasting blood glucose, such as diabetes or pre-diabetes, experience significant blood glucose fluctuations due to carbohydrate intake [40]. These fluctuations can impact brain energy supply, metabolism, and cognitive functions, notably memory performance.

Our study revealed that both an HCD and HFD induced abnormal glucose metabolism in db/db mice, resulting in elevated fasting blood glucose levels, impaired glucose tolerance, and insulin tolerance, and HOMA-IR results indicated that the HFD induced a greater degree of insulin resistance compared to the HCD. In the WT group, an HFD led to increased fasting glucose levels or impaired glucose tolerance, but an HCD did not. These findings guided our subsequent investigation, where fasting blood glucose levels were comparable between the HCD and HFD subgroups, enabling us to discern which diet had a greater impact on cognitive impairment. In agreement with our results, Brandon et al. reported that an HCD did not lead to impaired glucose tolerance in normal C57BL6/J mice, but their study did not include db/db mice [41]. O W Rasmussen et al. found HCD-induced blood glucose increase in non-insulin-dependent diabetes mellitus [42]. In contrast to our study, Chan-Hee Jung et al. reported that an HCD resulted in significant weight loss and a reduction in plasma glucose and HbA1c levels in patients with T2DM [43]. One possible explanation could be the increased intake of fiber, which can contribute to the feeling of satiety and lead to decreased consumption.

In addition, we examined the pathological changes in the hippocampus of mice. The hippocampus, being the primary functional region for learning and memory, exhibits heightened sensitivity to HFDs [44]. We investigated the effects of an HCD on hippocampal pathology, encompassing Aβ burden, tau protein hyperphosphorylation, and neuroinflammation. In accordance with prior previous research findings, an HFD induced increased hippocampal Aβ deposition, hyperphosphorylation of tau protein, and quantity of microglia and astrocytes, thereby triggering neuroinflammation in both WT and db/db mice [18,45]. However, the impact of an HCD on the two genotypes of mice varied. Firstly, in db/db mice, an HCD exacerbated hippocampal Aβ burden compared to an ND, albeit to a lesser extent than an HFD. A study conducted on healthy individuals aged 65 to 90 found a positive association between high intake of sugar and carbohydrates and global amyloid, but the study did not include diabetic patients [46]. Conversely, a low glycemic index of carbohydrates may improve Aβ burden [47]. Secondly, an HCD significantly increased the phosphorylation levels of tau protein at the Ser396 and Thr231 sites but to a lesser extent than that induced by an HFD. We also identified the essential role of the PI3K/Akt/GSK-3β signal pathway in HCD-induced cognitive impairment. The phosphorylation level of PI3K, Akt, and GSK-3β decreased, indicating the increased activity of GSK-3β and promoting the phosphorylation of tau protein. Hyperglycemia and elevated insulin levels could explain this phenomenon. Hyperphosphorylation of tau protein was also observed in Human Neurons-hippocampal (HN-h) cells exposed to different high glucose concentrations [48]. A prior study indicated that PS1, a protein involved in familial Alzheimer’s disease, suppressed GSK-3-dependent phosphorylation of tau by activating PI3K/Akt signaling and promoting phosphorylation of GSK-3 [35].

Overall, our research explored the impact of an HCD and an HFD on cognitive function in db/db mice compared to WT mice. Additionally, we conducted a comparison of the effects of both an HCD and an HFD on cognition under conditions where body weight and blood sugar levels were equivalent. The findings have significant implications regarding the impact of high-carbohydrate diets on cognition, which may indicate that individuals with diabetes should avoid high-carbohydrate diets also from a cognitive perspective. One limitation of this study is the lack of thorough investigation of the underlying mechanisms causing cognitive impairments and related pathological changes in db/db mice resulting from HCDs. The previous literature has reported both beneficial and detrimental effects of different carbohydrate compositions on blood glucose control in diabetic patients, with similar implications for cognition [49], but we did not identify the specific nutrient component in the HCD responsible for this effect. These limitations will be addressed in our future research.

## Figures and Tables

**Table 1:** Dietary pattern composition.

	Normal Diet		High-Carbohydrate Diet		High-Fat Diet	
Protein	25%		20%		20%	
Carbohydrate	55%		73%		20%	
Fat	20%		7%		60%	
Total	100%		100%		100%	
Ingredient						
	g/kg	kcal/kg	g/kg	kcal/kg	g/kg	kcal/kg
Casein	246.31	985.24	197.0	788.0	200.0	800.0
L-Cystine	3.6946	14.7784	3.0	12.0	3.0	12.0
Corn Starch	340.98	1363.92	454.65	1818.62	0	0
Maltodextrin	113.23	452.92	150.97	603.89	125.0	500.0
Sucrose	85.782	343.128	114.37	457.49	68.8	275.0
Cellulose	50.0	0	50.0	0	50.0	0
Soybean Oil	88.889	800.001	31.1	280	25.0	225.0
Lard	0	0	0	0	245.0	2205.0
Vitamin Mix V10037	10.0	40.0	10.0	40.0	10.0	40.0
Mineral Mix S10022G	35.0	0	35.0	0	10	0
Choline Bitartrate	2.5	0	2.5	0	2	0
Total	1048.6	4000.0	976.4	4000.0	773.85	4057

## Data Availability

The datasets used and/or analyzed during the current study are available from the corresponding author upon reasonable request due to legal or ethical reasons.

## Supplementary Materials

The following supporting information can be downloaded at: https://www.mdpi.com/article/10.3390/biomedicines12081701/s1, Figure S1: Representative images of IBA1 (microglia) costained with GFAP (astrocytes) in the hippocampus of mice in the WT group (scale bar = 200 µm).

## Abbreviations

HCD: high-carbohydrate diet; db/db: diabetic/diabetic; WT: wild-type; ND: normal diet; HFD: high-fat diet; T2D: type 2 diabetes; AD: Alzheimer’s disease; Aβ: amyloid β; GI: glycemic index; SPF: specific pathogen-free; GTT: glucose tolerance test; ITT: insulin tolerance test; TG: triglyceride; TC: total cholesterol; RT-qPCR: quantitative reverse transcriptase-PCR; GAPDH: glyceraldehyde 3-phosphate dehydrogenase; ELISA: enzyme-linked immunoassay; HOMA-IR: Homeostatic Model Assessment of Insulin Resistance; HbA1c: hemoglobin A1c; APP: amyloid precursor protein; BACE1: β-site APP cleaving enzyme-1; PI3K: phosphatidylinositol 3-kinase; Akt: protein kinase B; GSK-3β: Glycogen synthase kinase-3β; IBA1: ionized calcium-binding adaptor molecule 1; GFAP: glial fibrillary acidic protein; HN-h: Human Neurons-hippocampal; IL-1β: interleukin-1β; IL-6: interleukin-6; TNF-α: tumor necrosis factor-alpha.
