# Supplementary figures and images for: A High-Carbohydrate Diet Induces Cognitive Impairment and Promotes Amyloid Burden and Tau Phosphorylation via PI3K/Akt/GSK-3β Pathway in db/db Mice
Source: Biomedicines. 2024 Jul 31;12(8):1701. doi: 10.3390/biomedicines12081701 (PMC11351503; doi:10.3390/biomedicines12081701)

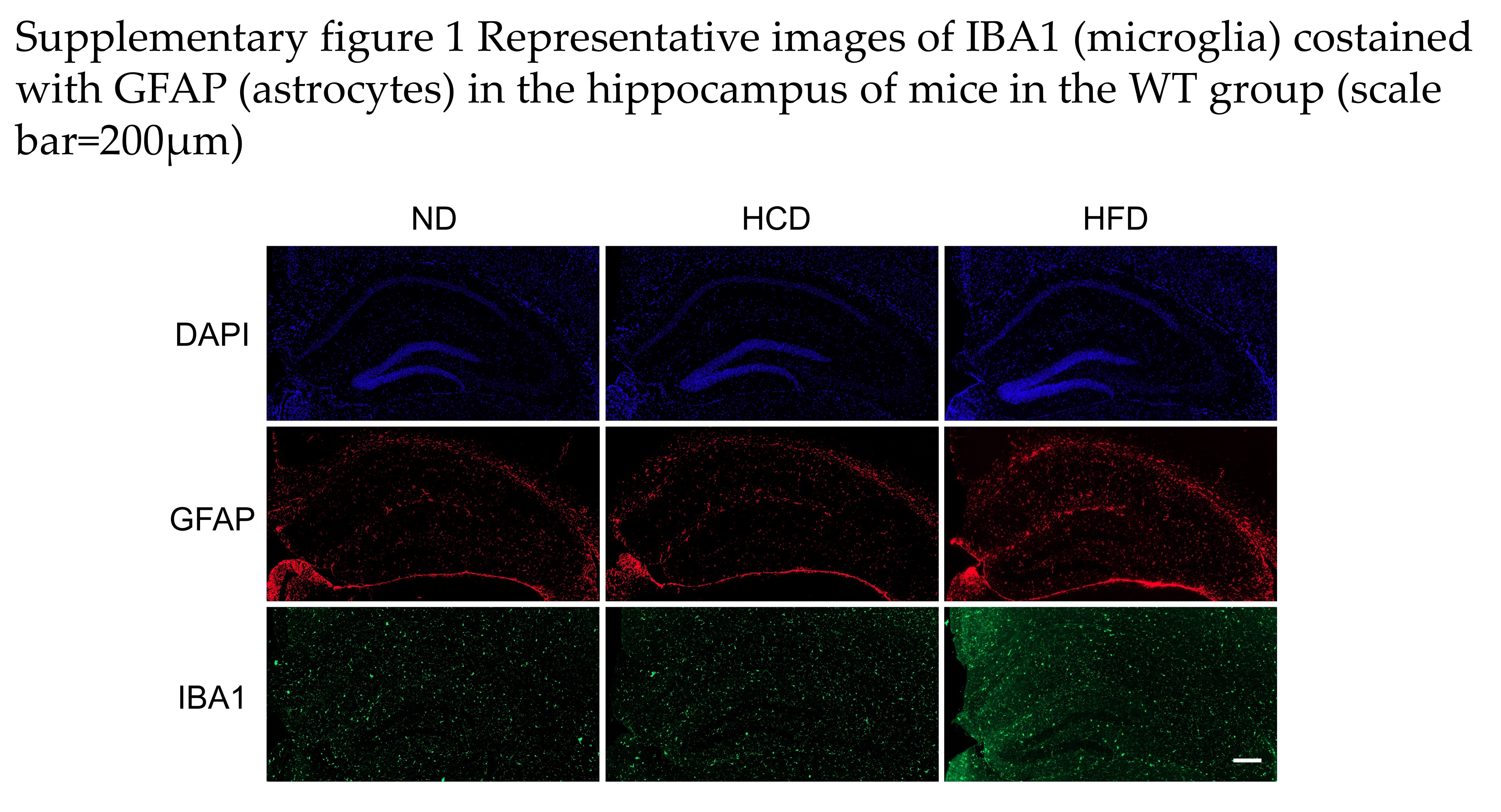

Supplement: Supplementary file 1 [file biomedicines-12-01701-s001.zip › biomedicines-3089712-supplementary.jpg]
